# Transient Exposure to Hypoxic and Anoxic Oxygen Concentrations Promotes Either Osteogenic or Ligamentogenic Characteristics of PDL Cells

**DOI:** 10.1089/biores.2014.0049

**Published:** 2015-02-01

**Authors:** Takako Kawasaki, Yoshinori Sumita, Kazuhiro Egashira, Seigo Ohba, Hideaki Kagami, Simon D. Tran, Izumi Asahina

**Affiliations:** ^1^ Department of Regenerative Oral Surgery, Unit of Translational Medicine, Graduate School of Biomedical Sciences, Nagasaki University, Nagasaki, Japan.; ^2^ Laboratory of Craniofacial Tissue Engineering, Faculty of Dentistry, McGill University, Montreal, Canada.; ^3^ Department of Oral and Maxillofacial Surgery, Matsumoto Dental University, Shiojiri, Japan.

**Keywords:** periodontal ligament, mesenchymal stem cells, cell culture, oxygen concentration, tissue regeneration

## Abstract

The periodontal ligament (PDL) has a reservoir of mesenchymal stem cells (MSCs) and this tissue is easily available following teeth removal procedures. However, PDL-derived cells (PDLCs) availability for tissue engineering is limited because they are heterogeneous cells at various differentiation and lineage commitments. Therefore, efficient culture conditions to increase MSCs number are needed to use PDLCs in tissue engineering. Recent reports indicate that low-oxygen conditions amplified stem/progenitor cell numbers and inhibited cell differentiation. Our aim was to establish which low-oxygen culture conditions favored bone or tendon/ligament regeneration in cultured PDLCs. Human PDLCs were cultured and exposed to either hypoxic (O_2_≤5%) or anoxic (O_2_<0.1%) oxygen conditions in low-glucose/serum-free media for 24 hours. After 24 h, as expected, cell survival was significantly less in PDLCs exposed to anoxic conditions as compared with cells under normal or hypoxic conditions. PDLCs exposed to hypoxic conditions had the highest percentages for MSC markers (CD105, CD166, Stro-1). For both hypoxic and anoxic conditions, stem cell marker genes (*oct4*, *sox2*, *p75*) were upregulated after 6 h. At 24 h, these stem cell markers were maintained in PDLCs under hypoxic condition. Interestingly under anoxic conditions, expression of *scleraxis* gene (a key transcription factor for tendo/ligamentogenesis) was upregulated markedly. When hypoxic PDLCs were subcultured into osteogenic medium, *in vitro* calcification and prominent *in vivo* bone formation in mice calvaria were observed. When anoxic PDLCs were subcultured into tendo/ligamentogenic medium, expression of *aggrecan* (a mature tenogenic gene) increased remarkably. No obvious differences were detectable on chondrogenic and adipogenic inducibilities. We propose that transient exposure to low-oxygen during the culture enhanced MSC population in PDL. In addition, different low-oxygen concentrations favored osteogenic or tendo/ligamentogenic inducibilities of cultured PDLCs.

## Introduction

Periodontal ligament-derived cells (PDLCs) have been proposed as a cellular reservoir of mesenchymal stem cells (MSCs) for bone and periodontal tissue engineering because these cells can supply mineralized dental tissues such as alveolar bone and cementum.^1,2^ For dentists, periodontal ligament (PDL) tissues can easily be harvested from common teeth removal procedures without morbidity, and thus are a convenient source of MSCs for oral/maxillofacial tissue reconstruction. However, their availability for tissue engineering is despairingly limited because even healthy extracted teeth, such as third molars, may retain only a limited amount of PDL tissue. Moreover, there are reports that the osteogenic potential of PDLCs is inferior to that of bone marrow MSCs (BMMSCs) or dental pulp–derived cells (DPCs).^3,4^ The two reasons are that (1) native PDLCs have both an osteogenic and a tendo/ligamentogenic characteristic, and (2) cultured PDLCs are composed of a heterogeneous population of cells at various stages of differentiation and lineage commitment compared to BMMSCs and DPCs.^5–7^ Therefore, to obtain a sufficient number of potential PDL stem/progenitor cells for tissue engineering, an expansion process of these cells is required *in vitro*. However, the reality is that PDLCs lose their plasticity severely during culture expansion and cell passaging.^7^ Thus simple and efficient culture conditions to obtain a substantial number of MSCs (that are maintained in an immature state of differentiation) are needed to translate the use of PDLCs for tissue engineering into a clinical reality.

Cell differentiation is regulated by several key factors, such as growth factors, transcription factors, cell-to-matrix and cell-to-cell interactions, and as reported more recently, by oxygen tension.^8^ Low-oxygen conditions are considered as one of the key factors in maintaining an undifferentiated state and plasticity of somatic stem cells.^9^ In fact, the native environment for most somatic stem cells is hypoxic (e.g., 1%–7% oxygen [O_2_] in bone marrow).^9,10^ BMMSCs reside in a self-renewing state at low *p*O_2_ and tend to differentiate toward functional osteoblasts when located closer to blood vessels and exposed to higher *p*O_2_.^9^ Meanwhile, *in vitro*, conventional cell culture is performed under ambient oxygen (21%) condition. Recent studies have indicated that low-oxygen conditions, such as hypoxic or anoxic culture conditions, amplified stem/progenitor cell numbers, and inhibited cell differentiation.^11^ For example, hypoxic stimulation with 3% O_2_ inhibited differentiation of BMMSCs and enhanced activation of pluripotent stem cell markers.^11^ These facts suggest that hypoxic/anoxic stimulations to dental MSCs in culture can arrest their differentiation and enhance their plasticity. Indeed, more recently, it has been reported hypoxic stimulation plays an important role in maintaining the stemness and differentiation capacity of PDLCs *in vitro*.^12^ However, for tissue engineering use, it has not been well ascertained what culture condition of PDLCs with low-oxygen is effective to obtain a reliable number of functional MSCs.

As a first step to establish the concrete conditions of cell culture with low-oxygen stimulation for tissue engineering, the aim of this study is to determine whether hypoxic or anoxic conditions are optimal for bone or tendon/ligament regeneration of PDLCs. In this study, transient (short-time) exposure to low-oxygen conditions in culture was investigated to enhance the regenerative capability of PDLCs. Previously, we demonstrated upregulation of *oct4* and *sox2* genes in surviving DPCs after short-time (6 to 24 hours) exposure of anoxic conditions (O_2_<0.1%).^13^ Short-time treatment with low-oxygen can be easily applied to cells during the culture process for future clinical use. Therefore, we hypothesized that short-time exposure of the proper concentration of low oxygen may enrich the subpopulation of PDL stem/progenitor cells or enhance the plasticity of cultured PDLCs even at later passages in culture. Here, we analyzed stem cell properties and regenerative capabilities of surviving PDLCs post exposure to transient low-oxygen conditions such as either hypoxic (O_2_≤5%) or anoxic (O_2_ 0.1%) conditions.

## Materials and Methods

### Preparation of PDLCs

The procedure used to harvest the extracted teeth from humans conformed to tenets of Declaration of Helsinki, and protocol was approved by Ethics Committee of Nagasaki University. All subjects provided written informed consent. Human periodontal ligament tissues were harvested from healthy normal third molars (5 patients, 3 males and 2 females; average 31years old) after tooth extraction. PDL tissue was mechanically removed by a scalpel, and a single-cell suspension was obtained enzymatically by digesting with 2 mg/mL collagenase (Worthington Biochem) for 1 hour in Dulbecco's modified Eagle's medium (DMEM; Cat No. 041-29775, Sigma) containing 10% fetal bovine serum (FBS; Thermo Trace Ltd) and 2 % antibiotic–antimycotic solution on a shaker at 37°C. Isolated cells were cultured in medium (DMEM containing 10 % FBS and 2% antibiotic–antimycotic solution). When cells reached 80%–90 % confluence, they were subcultured until passage 6 (P6) (population doubling level 24) for subsequent experiments.

### Low-oxygen exposure

Low-oxygen conditions for cell culture were achieved by use of an anaerobic jar and AnaeroPack (Mitsubishi Gas Chemical) for hypoxia (5% O_2_) and anoxia (0.01% O_2_). For experiments, P5–P6, PDLCs were seeded at concentration of 5×10^4^ cells/well into 24-well plates (Thermo Fischer Scientific, Roskilde) and were cultured until 80%–90% confluence. Then, they were exposed to hypoxic/anoxic conditions in low-glucose/nonserum culture medium (DMEM containing 2% antibiotic–antimycotic solution) for 24 hours. Regarding this condition, we performed preliminary experiments to determine the optimum concentrations of glucose and exposure time of cells to low-oxygen as described in our previous work.^13^ Briefly, cell viability was measured at three different concentrations (non, 0 g/L; low, 1 g/L; and high, 4.5 g/L) in normoxic conditions to determine the glucose concentration, and it was also examined in hypoxic/anoxic conditions every 6 h within 48 h to determine the exposure time for low-oxygen. As a result, low glucose concentration was selected for this study, as it was effective for cell viability under serum-free and normoxia conditions when compared with nonglucose or high-glucose concentrations (data not shown). For the length of exposure to low-oxygen tensions, almost all cells died in anoxic conditions after 36 hours, while more than 50 % of cells could survive within 24 hours (data not shown). Therefore, the evaluations of this study were performed mainly at 24 hours as transient low-oxygen exposures, except the gene expression analysis of stem cell markers at 6 hours (as an early stage after established low-oxygen conditions). To analyze the effects of low-oxygen stimulations, experiments were performed in each experimental group: control (Contl), cells in DMEM with low glucose/10% FBS under 21% O_2_ normoxia; normoxia (Normo), cells in low-glucose/nonserum DMEM with under 21% O_2_ normoxia for 24 hours; hypoxia (Hypo), cells in low-glucose/nonserum DMEM with under 5% O_2_ hypoxia for 24 hours; and anoxia (Ano), cells in low-glucose/nonserum DMEM with under 0.01% O_2_ anoxia for 24 hours.

### Cell viability

Cell number and cell viability was assessed by WST-8 kit and Double-Staining Kit (Dojindo, Kumamoto, Japan) according to manufacturer's protocol. Calcein-AM stains viable cells, while propidium iodide (PI) only stains the nuclei of dead cells. They were evaluated at 24 hours after low-oxygen exposure. Each experiment was performed in triplicate for three samples.

### Protein expressions

Flow cytometry experiments used a FACscan argon laser cytometer (BD Biosciences) using fluorescein isothiocyanate (FITC) or phycoerythrin (PE) conjugated monoclonal antibodies to CDs 34, 45, 90, 105, 146, 166, and 271 (each 1:100) (Becton) and Stro-1 (1:400) (R&D); 7-amino-actinomycin D (7-AAD) (Cayman Chemicl) was used to detect dead cells. After cultured PDLCs were detached with Trypsin-EDTA, cells were then incubated with individual antibodies for 20 min on ice. In case of labeling with Stro-1 antibody, cells were further incubated with RHODAMINE-conjugated goat (1:50) (Millipore) anti-mouse secondary antibodies for 1 h at room temperature. Finally, the cells were washed, resuspended in 200 μL of ice-cold phosphate buffered saline, stained with 7-AAD for 5 minutes, and analyzed.

To confirm the presence of Vimentin, Stro-1, and hypoxia-inducible factor-1 alpha (HIF1α) proteins in PDLCs, immunocytochemistry was performed. After blocking for 30 min, cells were incubated with mouse monoclonal anti-human Vimentin (1:50) (Dako), Stro-1 (1:400) (Millipore,), or HIF1α (1:100) (R&D) for 1 h at room temperature. The slides were then incubated with FITC-conjugated donkey (1:200) (Jackson) or RHODAMINE-conjugated goat (1:100) (Millipore) anti-mouse secondary antibodies for 1 h at room temperature. Control staining was performed by replacing the first antibody with preimmune serum eluted from the corresponding affinity columns.

### Gene expressions

Reverse transcription polymerase chain reaction (RT-PCR) and quantitative PCR were used to determine the mRNA expressions of stem cell (*oct4*, *sox2*, *p75ntr*) and osteogenic (*runx2*, *ocn*) / tenogenic (*scleraxis*, *aggrecan*) / chondorgenic (*sox9*, *decorin*) / adipogenic (*pparγ*, *lpl*) differentiation markers. Total RNA was extracted with Trizol-reagent (Invitrogen), First-strand complementary DNA synthesis was performed by SuperScript First-Strand Synthesis (Invitrogen). Complementary DNA was amplified with Takara-Taq (Takara Bio Inc.). PCR reactions were performed with Mx3000P QPCR System (Agilent Technologies). Human-specific primer sets are shown in Table 1. As internal standards, glyceraldehyde-3-phosphate dehydrogenase (*gapdh*) and hypoxanthine phosphoribosyl transferase 1 (*hprt1*) primers were used for RT-PCR and quantitative PCR, respectively.

**Table 1.** **Sequence of Primer Pairs Used for RT-PCR and Quantitative PCR**

*Gene*	*Sense primer*	*Antisense primer*
*oct4*	5′-CACTGTACTCCTCGGTCCCTTTC-3′	5′-CAGGCACCTCAGTTTGAATGC-3′
*sox2*	5′-GTGAGCGCCCTGCAGTACAA-3′	5′-GCTGCGAGTAGGACATGCTGTAG-3′
*p75ntr*	5′-CGACAACCTCATCCCTGTCT-3′	5′-CTGTTGGCTCCTTGCTTGTT-3′
*runx2*	5′-CCTGAACTCTGCACCAAGTC-3′	5′-CATCGGTGATGGCAGGAAGC-3′
*scleraxis*	Hs SCXB 2 SG QuantiTect primmer (QIAGEN)	
*pparγ*	5′-CTCCTATTGACCCAGAAAGC-3′	5′-GTAGAGCTGAGTCTTCTCAG-3′
*sox9*	5′-ACACACAGCTCACTCGACCTTG-3′	5′-GGAATTCTGGTTGGTCCTCTCTT-3′
*ocn*	5′-CAAAGGTGCAGCCTTTGTGTC-3′	5′-TCACAGTCCGGATTGAGCTCA-3′
*aggrecan*	5′-GTGCCTATCAGGACAAGGTCT-3′	5′-GATGCCTTTCACCACGACTTC-3′
*lpl*	5′-ATGGAGAGCAAAGCCCTGCTC-3′	5′-GTTAGGTCCAGCTGGATCGAG-3′
*decorin*	5′-CGCCTCATCTGAGGGAGCTT-3′	5′-TACTGGACCGGGTTGCTGAAA-3′
*gapdh*	5′-GGACTCCACTGGCGTCTTCAC-3′	5′-GCTGATGATCTTGAGGCTGTTGTC-3′
*hprt1*	5′-TGACACTGGCAAAACAATGCA-3′	5′-GGTCCTTTTCACCAGCAAGCT-3′

### Induction and evaluation after reoxygenation

After receiving oxygen stimulations for 24 hours, cells were exposed to inductive media under normoxia. For osteogenic differentiation, reoxygenated cells were seeded at concentration of 5×10^4^ cells/well into 24-well plates (Thermo) and cultured with 10^–5^ M dexamethasone (3.92×10^–2^ g/L), 10 mM β-glycerophosphate (2.16 g/L), and 50 mM ascorbate-phosphate (8.8 g/L) (Sigma). After 2 weeks, cells were stained with 40 mM of Alizarin-red sulfate (pH 4.2) (Sigma) to assess *in vitro* calcification as described in our previous works.^14,15^ For tendo/ligamentogenic differentiation, reoxygenated cells were seeded at concentration of 2.5×10^4^ cells/well onto 12-well plates (Thermo) and cultured with 10 ng/mL recombinant mouse (rm) growth and differentiation factor 5 (R&D) containing 1% fetal bovine serum. After 1 week, expression of *aggrecan* (a marker of tenocytic differentiation) mRNA in cultured cells was evaluated to assess the capability of tendo/ligamentogenesis. Although this gene is a marker of chondrogenic differentiation, it is known that its expression is also upregulated in tenocytic cells with increased expression of *scleraxis*.^7,16^ For chondorogenic differentiation, reoxygenated cells were seeded at concentration of 1×10^6^ cells into a 15-mL tube, and cell pellets were cultured in chondrogenic-induction medium (Cat No. PT-3003) supplemented with transforming growth factor β3 (10 ng/mL) (Lonza) for 4 weeks. After 4 weeks, the sections of pellets were stained with 0.1 g of toluidine blue in 100 mL of distilled water (pH 4.1) (Sigma). For adipogenic differentiation, reoxygenated cells were seeded at concentration of 5×10^4^ cells/well into 24-well plates (Thermo) and cultured in adipogenic-induction medium (Cat No. PT-3004, Lonza) according to manufacturer's protocol. After 3 weeks, intracellular lipid accumulation was visualized using fresh Oil Red O staining.

### *In vivo* *experiment*

Animal experiments were performed at the Animal Center of Nagasaki University, and Guidelines for Animal Experimentation were observed. After 2 weeks of osteogenic induction *in vitro*, harvested cells in each group (1×10^6^ cells/group) were mixed with 20 mg of β-tricalcium phosphate (β-TCP) granules (0.5–1.5 mm size; Osferion®, Olympus). Before 24 h of mixing, 0.5 μg of recombinant human (rh) bone morphogenic protein (BMP) 2 (donated by Astellas Pharma Inc.) in 40 μL solution were adsorbed to 20 mg β-TCP granules, and then these materials were lyophilized in advance. Before transplantation, 10 mL bovine thrombin and 10% calcium chloride mixture (1:1 ratio) was added to the β-TCP/cell mixture to trigger fibrin polymerization to produce an insoluble gel, then these constructs were transplanted to the onlay placement of calvaria of 6-week-old BALB/cAJcl-nu/nu mice (Nihoncrea). After 2 weeks of transplantation, the specimens were harvested. For histological observation, harvested specimens were fixed in 4% paraformaldehyde; decalcified with a solution containing 2.9% citric acid, 1.8% trisodium citrate dehydrate, 10% formic acid, and 90% distilled water; and embedded in paraffin wax. Then, sections (3 μm) were deparafinized and stained with hematoxylin and eosin (HE). Regarding the animal model in this study, we performed a preliminary experiment to determine optimal dose of rhBMP2. We transplanted 20 mg β-TCP granules with various concentrations (0, 0.1, 0.5, and 1 μg) of rhBMP2 onto the calvaria of mice. As a result, rhBMP2 at concentrations of 0.5 and 1 μg could induce the new bone formation after 2 weeks of transplantation (data not shown). However, bone formation by 0.5 μg rhBMP2 was minimal, while abundant bone formation was detectable in specimens of 1μg rhBMP2. On the other hand, more abundant bone formation was recognized in specimens of both concentrations after 4 weeks (data not shown). Therefore, we considered 0.5 μg of rhBMP2 is optimal to find the effect of this experiment on 2 weeks of transplantation in this study.

### Statistical analysis

Means were analyzed using one-way analysis of variance. Dunnett's multiple comparison *t*-test was used to detect any significant differences within each group. Experimental values were presented as mean±standard deviation A *p*-value <0.05 was considered to be statistically significant.

## Results

### Characteristics of PDLCs

**FIG. 1.**
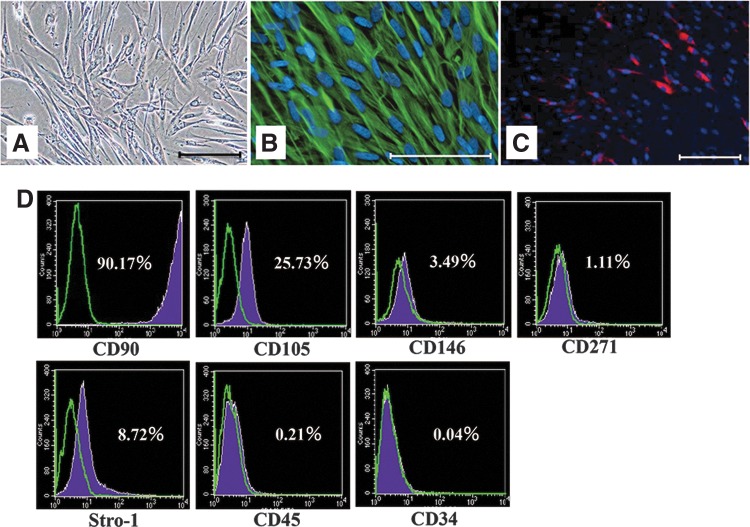
Cell characterization of cultured periodontal ligament-derived cells (PDLCs) at passage 5. **(A)** PDLCs showed a characteristic spindle shape in Dulbecco's modified Eagle's medium with 10% fetal bovine serum. Scale bar is 100 μm (100×). **(B)** Immunofluorescence staining of anti-Vimentin antibody. This mesenchymal cell marker is labeled by fluorescein isothiocyanate (green); nuclei are labeled with DAPI (blue). Scale bar is 100 μm (200×). **(C)** Immunofluorescence staining of anti-Stro-1 antibody. This stem cell marker (labeled by phycoerythrin) (red) is detected in some cells; nuclei are labeled with DAPI (blue). Scale bar is 100 μm (100x). **(D)** Flow cytometric analysis of mesenchymal stem cell markers (CD90, CD105, CD146, CD271, Stro-1) and hematopoietic cell markers (CD34, CD45) for passage 5 (P5) PDLCs.

P5 PDLCs had a characteristic spindle shape and formed a monolayer (Fig. 1A). Immunofluorescence analysis revealed that human anti-Vimentin (mesenchymal cell marker) antibody reacted with cultured PDLCs (Fig. 1B), and anti-Stro-1 (multilineage stem/progenitor marker) antibody reacted with some cells (Fig. 1C). Cell surface markers on P5 PDLCs were characterized using immunofluorescence combined with flow-cytometric analysis. Many cells were positive for CD90 (90.17%) (mesenchymal cell marker), and there was a low level of CD 105 (25.73%), CD146 (3.49%), CD 271 (1.11%), and Stro-1 (8.72%) (MSC markers). In contrast, CD34 and CD45 (hematopoietic cell markers) were almost absent (Fig. 1D).

### Cell viability

**FIG. 2.**
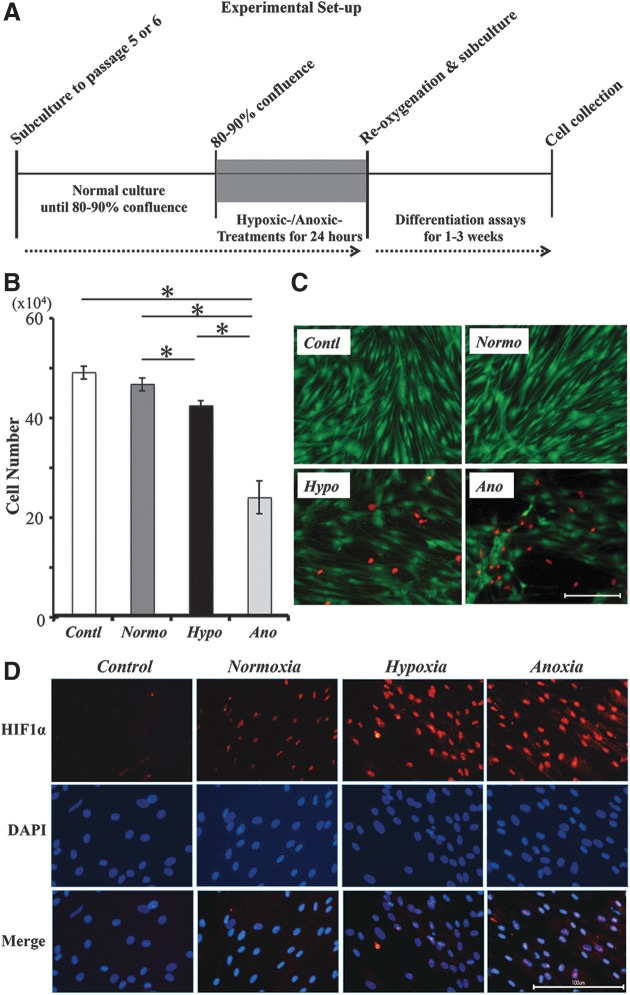
Low-oxygen stimulation to PDLCs during the culture. **(A)** Schema of the experimental set-up. **(B)** Number of surviving cells was decreased significantly under hypoxic/anoxic conditions. Experiment was performed in triplicate for three samples. The asterisk represents statistical significance (*p*<0.05). **(C)** Many dead cells were detected in cells exposed to hypoxic/anoxic conditions. In contrast, no dead cells were observed under normoxic conditions. Scale bar is 100 μm (100×). To visualize the live (green) and dead (red) cells, double staining with Calcein-AM and propidium iodide was performed. **(D)** Number of surviving cells under hypoxic/anoxic conditions after 24 hours expressed HIF1α compared with cells under normoxia. Scale bar is 100 μm (200×). *Contl*, PDLCs cultured in normal culture medium under normoxia; *Normo*, PDLCs cultured in low-glucose/serum-free medium under normoxia for 24 hours; *Hypo*, PDLCs cultured in low-glucose/serum-free medium under hypoxia for 24 hours; *Ano*, PDLCs cultured in low-glucose/serum-free medium under anoxia for 24 hours.

We first analyzed the effect of transient low-oxygen stimulations (hypoxic/anoxic cultures for 24 hours) on cell viability. When P5–P6 PDLCs reached 80%–90 % confluence, they were exposed to hypoxic or anoxic culture conditions in low-glucose/serum-free media (Fig. 2A). After 24 hours, the number of surviving cells decreased significantly under hypoxic/anoxic conditions (Fig. 2B). In particular, only approximately 50% cells survived under anoxic conditions. To visualize live and dead cells, double staining with Calcein-AM and PI was performed. The dead cells were observed in cells exposed to hypoxic/anoxic conditions, obviously, though many dead cells had been already detached from the dish (Fig. 2C). In contrast, no dead cells were detected under normoxic conditions. Moreover, a higher number of surviving cells under hypoxic/anoxic conditions expressed HIF1α when compared with cells under normoxia (Fig. 2D).

### Characteristics of surviving cells after stimulations

**FIG. 3.**
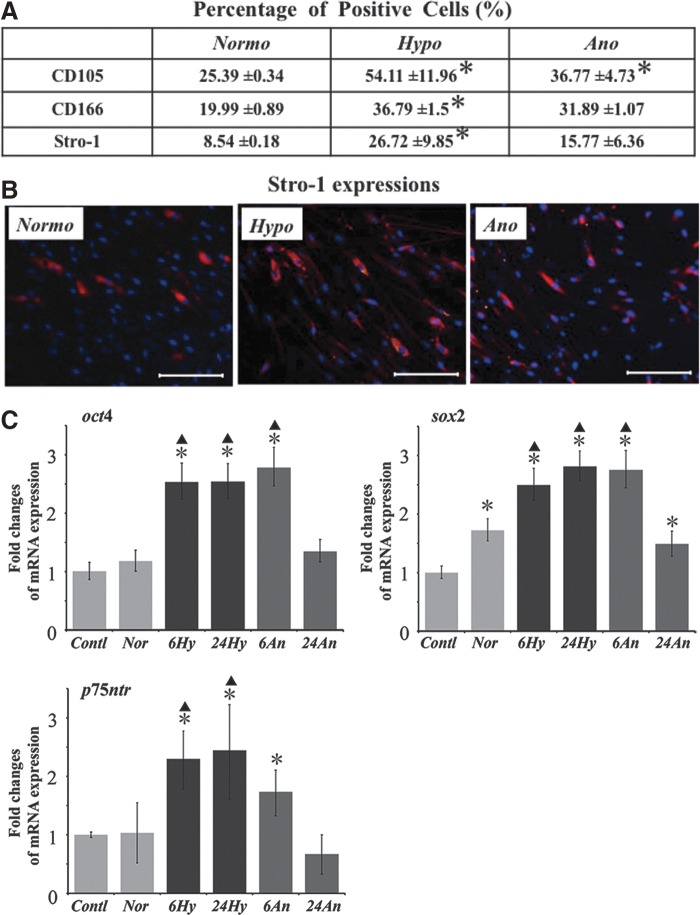
Stem cell characteristics of surviving cells after transient low-oxygen tensions. **(A)** Flow-cytometric analysis of positive cells for stem cell markers (CD105, 166 and Stro-1) was examined after 24 hours of stimulation. Percent positivity (%) is displayed as mean±standard deviation. All analyses were performed on five donors. *Normoxia group versus hypoxia or anoxia groups (*p*<0.05). **(B)** Changes of Stro-1 expression in PDLCs after low-oxygen stimulations. Scale bar is 100 μm (100×). The expression was highest in cells survived under hypoxic-condition. **(C)** Quantitative real-time polymerase chain reaction analysis of stem cell marker genes (*oct4*, *sox2*, and *p75ntr*). *Contl*, the cells cultured in normal culture medium under normoxia; *Nor*, the cells cultured in low-glucose/serum-free medium under normoxia for 24 hours; *6Hy* or *24Hy*, the cells cultured in low-glucose/serum-free medium under hypoxia for 6 or 24 hours; *6An* or *24An*, the cells cultured in low-glucose/serum-free medium under anoxia for 6 or 24 hours. *Contl group versus experimental groups (*p<0.05). ^▲^Normoxia group versus hypoxia or anoxia groups (^▲^*p*<0.05).

Low-oxygen stimulations increased the proportion of cells positive with stem cell markers. The percentage of MSC markers (CD105, 166 and Stro-1) was higher in cells that survived in both low-oxygen conditions than those in normoxia (Fig. 3A, B). Particularly, the percentage of these markers was significantly higher in cells that survived in hypoxic conditions. Moreover, the changes in gene expression of pluripotent stem cell markers were investigated at 6 hours (as an early stage after established low-oxygen condition) and 24 hours of hypoxic/anoxic conditions. The expression of these markers (*oct4*, *sox2,* and *p75ntr*) was upregulated markedly in PDLCs under 6–24 hours hypoxic and 6 hours anoxic conditions (Fig. 3C). However, these expressions were decreased in surviving cells under anoxic conditions at 24 hours.

### Reoxygenation and differentiation of surviving cells

**FIG. 4.**
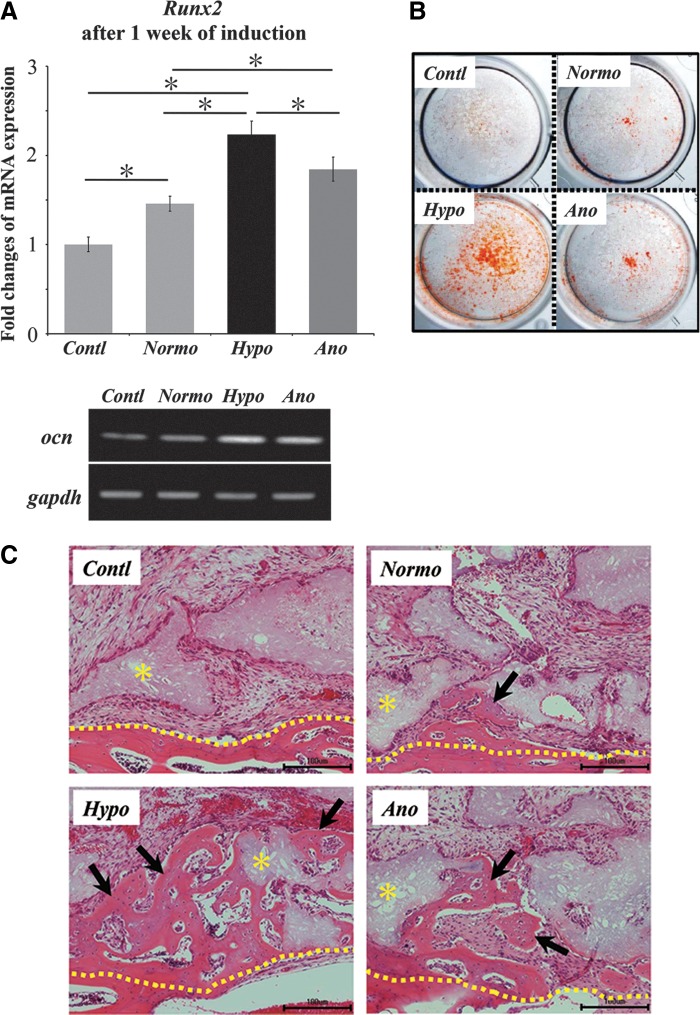
Osteogenic differentiation of surviving cells after reoxygenation. **(A)** mRNA expressions of *runx2* (a transcription factor of osteogenic differentiation) and *osteocalcin* (*ocn*, a marker of osteogenic-differentiation) after 1 week of osteogenic induction. The expressions of these genes were upregulated in cells exposed to low-oxygen conditions. **(B)** Alizarin red stainin after 2 weeks of induction. Positive nodules were detected markedly in cells exposed to hypoxic-condition. **(C)** Bone formation after 2 weeks of transplantation. Scale bar is 100 μm (100×). The cells after 2 weeks of induction were transplanted to mouse calvaria. New bone formation was promoted in specimens of hypoxic treatment. Black arrow, new bone area; yellow asterisk, β-tricalcium phosphate granules; yellow-dotted line, boundary of new bone and calivaria bone.

After PDLCs were exposed to low-oxygen conditions for 24 hours, surviving cells were collected and reoxygenated (O_2_=21%) to assess capability of multilineage differentiation. Reoxygenated cells were subsequently cultured under the condition of osteogenic/tenogenic/chondrogenic/adipogenic differentiations. When cells were cultured in osteogenic medium for 1 week, increased expressions of *runx2* gene (a transcription factor of osteogenic differentiation) were found in cells exposed to low-oxygen conditions (Fig. 4A). In particular, this expression is highest in cells that survived under hypoxic conditions. Furthermore, expressions of *osteocalcin* (a marker of osteogenic differentiation) mRNA were upregulated in cells exposed to low-oxygen conditions. Then, after 2 weeks, cells exposed to hypoxic conditions formed alizarin red–positive nodules markedly (Fig. 4B). Moreover, when cells after 2 weeks of osteoblastic induction were transplanted to mouse calvaria, promoted bone formation was found on the histological observation (HE staining) in specimens of low-oxygen conditions on 2 weeks of transplantation (Fig. 4C). Particularly, cells exposed to hypoxic conditions could induce the abundant bone formation *in vivo*.

**FIG. 5.**
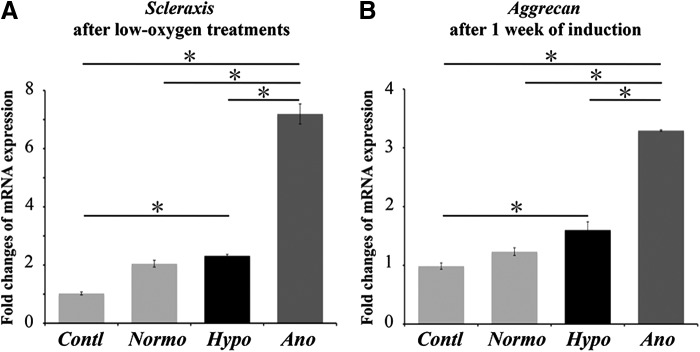
Tendo/ligamentogenic differentiation of surviving cells after reoxygenation. **(A)** Expression of *scleraxis* (a key transcription factor of tenocytic differentiation) just after 24 hours of low-oxygen stimulations. **(B)** Expression of *aggrecan* after 1 week of tenodo/ligamentgenic induction. Tenocytic transcription factor and tenocytic differentiation marker were upregulated markedly in cells exposed to anoxic conditions. All analyses were performed on three donors. The asterisk represents statistical significance (*p*<0.01).

On the other hand, we found *scleraxis* (a transcription factor of tenodon/ligament differentiation) expression was upregulated markedly in surviving PDLCs just after anoxic treatment (Fig. 5A). At the same time point, expression of runx2 (a transcription factor of osteogenic differentiation), sox9 (a transcription factor of chondrogenic differentiation), or *pparγ* (a transcription factor of adipogenic differentiation) genes has not shown obvious changes (data not shown). When cells were subsequently cultured in tendo/ligamentogenic medium for 1 week after reoxygenation, *aggrecan* (a marker of tenogenic differentiation) expression in cells exposed to anoxic conditions increased markedly (Fig. 5B).

**FIG. 6.**
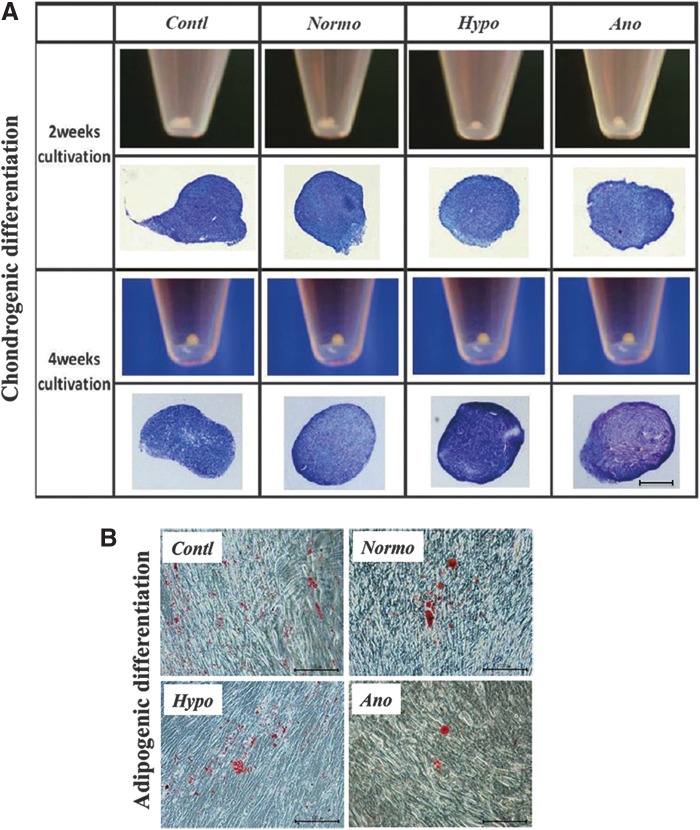
Chondrogenic/adipogenic differentiation of surviving cells after reoxygenation. **(A)** Gross appearance, toluidine blue staining of cell aggregates at 2 or 4 weeks of pellet cultivation for chondrogenic-differentiation. Scale bar is 500 μm (40×). Cells survived under hypoxic/anoxic conditions could form typical round-shaped pellets at 2 weeks of cultivation. After 4 weeks, toluidine blue staining demonstrated that most of the cells in pellets under hypoxic/anoxic conditions were surrounded by proteoglycan. **(B)** Oil red-O-staining after 3 weeks of adipogenic induction. Scale bar is 100 μm (100×). The difference of inducibility was not obvious among groups.

In chondrogenic differentiation medium, surviving PDLCs under hypoxic/ anoxic conditions formed typical round-shaped pellets at 2 weeks of cultivation (Fig. 6A). After 4 weeks, toluidine blue staining demonstrated that most cells in PDLCs pellets were surrounded by proteoglycan. These cells seemed to differentiate into chondrocyte-like cells. Meanwhile, adipogenic induction was not observed clearly in most cultured cells, and only a limited number of oil red O–positive lipid clusters were identified after 3 weeks (Fig. 6B). As a result, no obvious differences were detectable on chondrogenic and adipogenic inducibilities among experimental groups. Similarly, gene expression analysis has not shown obvious expression changes of *decorin, aggrecan* (markers of chondrogenic differentiation), or *lpl* (a marker of adipogenic differentiation) among each group after 4 weeks of chondrogenic or 3 weeks of adipogenic induction (data not shown).

## Discussion

This study demonstrated that short-time low-oxygen stimulation has an effect on tissue regenerative capability of cultured PDLCs. Our outcomes were (1) when receiving transient low-oxygen stimulations after confluence, surviving PDLCs showed an increased expression of MSC factors; (2) PDLCs that survived in low-oxygen conditions displayed an increased capacity for osteogenic and tendo/ligamentogenic differentiation after reoxygenation; and (3) these inducibilities might depend on the concentrations (hypoxia or anoxia) of low-oxygen. These outcomes suggest that transient low-oxygen stimulation during culture is a useful option to enrich immature dental MSC subpopulations, which can promote bone and tendon/ligament regeneration.

Regarding the first outcome related to stem cell characteristics, several studies have demonstrated that hypoxic conditions can amplify the number of stem/progenitor cells and have an inhibitory effect on their differentiation capability. For example, it was recently demonstrated 3% O_2_ inhibited differentiation of BMMSCs and enhanced the activation of stem cell markers such as *rex1* or *oct4*.^11^ Moreover, our group found transient activation of *oct4* and *sox2* in DPCs surviving under anoxic conditions for 6–18 h.^13^ Also, these studies reported the involvement of HIF1 as a possible mechanism/pathway. Indeed, it has been reported that HIF1 activation may influence the expression of several key markers of stem cell such as CXCR4 or Oct4.^17^ Our data are consistent with these previous studies and showed that PDLCs exposed to low-oxygen stimulations for 24 hours expressed HIF1α protein remarkably. Therefore, transient stimulation with low-oxygen tensions seemed to maintain or induce an undifferentiation status in surviving PDLCs through this pathway. To support this hypothesis, we profiled protein expression after 24 hours of hypoxic/anoxic stimulations and found an increased number of immature dental MSCs. In particular, PDLCs exposed to hypoxic conditions for 24 hours contained a high number of cells positive for MSC markers. These dental MSCs (in PDL) were identified by cell surface antigens such as CD105, 166, or Stro-1, all of which were also markers used for BMMSCs,^18^ and these stem cells showed the ability to differentiate into multiple cell types.^6^ Moreover, it is known that these cells show the increased expressions of both markers for embryonic stem cell (such as Oct4, Sox2 or SSEA4) and neural-crest/immature MSCs (such as p75NTR).^19^ In fact, in our study, expression of these genes (*Oct4*, *Sox2*, and *p75ntr*) was increased by stimulations. Our data reliably indicated that transient low-oxygen stimulation could induce or enrich the immature dental MSCs subpopulation within a heterogeneous population of PDLCs at a late stage of culture. However, anoxic stimulation for 24 h decreased the expression of these genes. Either activation of crucial stem cell genes by low-oxygen stimulation may be temporary in PDLCs during culture, or anoxic stimulation may be harmful even for undifferentiated cells.

With regard to the plasticity after reoxygenation, PDLCs exposed to hypoxic conditions for 24 hours displayed an increased differentiation capacity to osteoblasts. To date, research using PDLCs as a cell source has mainly focused on bone engineering because PDLCs can be coaxed into mineralized tissues like cementum and alveolar bone, with some growth factors such as BMP2.^14^ However, it is suggested that osteogenic potential of PDLCs is inferior to that of BMMSCs or DPCs.^3,4^ Particularly, in culture, PDLCs have a tendency to downregulate their capacity of osteoblastic and tenocytic differentiation during several passages.^7^ Therefore, development of a simple and effective method to obtain a sufficient number of dental MSCs (which maintain their plasticity in culture) within PDLCs being expanded for several passages is a crucial step in making the use of PDLCs a clinical reality for regenerative therapy. In this study, when P5–P6 PDLCs were exposed to hypoxic conditions for only 24 hours, surviving cells could form abundant new bone tissues *in vivo* after reoxygenation. At least hypoxic PDLCs may effectively reduce the amount of rhBMP2 required to induce bone formation in the natural body. Recombinant human BMP2 is known to induce substantial swelling that may cause the obstruction of the airway when applied to oral and cervical areas, though some clinical studies have shown successful bone regeneration following the direct implantation.^20,21^ On the other hand, interestingly, we found PDLCs survived after anoxic stimulation could show an increased expression of *scleraxis*, a key transcription factor associated with tendo/ligamentogenic differentiation. Also, the inducibility to tendo/ligament differentiation seemed to be superior in cells exposed to anoxic conditions after reoxygenation. Oxygen level of native tendon/ligament tissues must be lower when compared with other vascular-rich tissues because these tissues are collagen-rich structures including only a few blood vessels.^22,23^ Therefore, it is reasonable to speculate that short-time exposure with severely low oxygen (anoxia) can enrich the capable MSCs for tendo/ligamentogenic differentiation. These facts suggest that this simple strategy is helpful to obtain higher number of dental MSCs from cultured PDLCs, particularly for bone and ligament tissue engineering.

In conclusion, our findings demonstrated that short-time exposure to low-oxygen conditions during culture can enhance the capability of human PDLCs, even at late cell passages, for bone and ligament tissue engineering. In addition, the concentration of low-oxygen affects the osteogenic or tendo/ligamentogenic inducibilities of PDLCs. However, the effect of transient low-oxygen was not obvious for chondorogenic or adipogenic differentiation from PDLCs in this study. Additional investigations are needed to understand the practical usefulness of transient low-oxygen stimulation for reliable tissue engineering.

## Abbreviations Used

AAD: 7-amino-actinomycin D
BMMSCs: bone marrow MSCs
BMP: bone morphogenic protein
DMEM: Dulbecco's modified Eagle's medium
DPCs: dental pulp–derived cells
FBS: fetal bovine serum
FITC: fluorescein isothiocyanate
HE: hematoxylin and eosin
HIF1α: hypoxia-inducible factor-1 alpha
MSC: mesenchymal stem cells
RT-PCR: reverse transcription polymerase chain reaction
P: passage
PDL: periodontal ligament
PDLCs: periodontal ligament-derived cells
PE: phycoerythrin
rhBMP: recombinant human bone morphogenic protein
β-TCP: β-tricalcium phosphate
